# Modeling in Early Stages of Technology Development: Is an Iterative Approach Needed? Comment on "Problems and Promises of Health Technologies: The Role of Early Health Economic Modeling"

**DOI:** 10.15171/ijhpm.2019.118

**Published:** 2019-12-01

**Authors:** Michael F. Drummond

**Affiliations:** University of York, York, UK.

**Keywords:** Innovation Policy, Innovation, Health Technology Assessment, Health Economic Modeling, Early Assessment

## Abstract

A recent paper by Grutters et al makes the case for early health economic modeling in the development of health technologies. A number of examples of the value of early modeling are given, with analyses being performed at different stages in the development of several non-drug health technologies. This commentary acknowledges the contribution of the paper by Grutters et al and argues for an iterative and integrated approach to early modeling, assessing the cost-effectiveness of the technology, the value of future research and the interaction with the manufacturer’s pricing and revenue expectations.

## Introduction

In their recent paper, Grutters et al^1^ discuss the role of early health economic modeling in making key decisions in the development of health technologies. Their observations are based on 32 early modeling analyses of non-drug technologies undertaken by a subsidiary group of a university hospital in the Netherlands. The analyses were all conducted as a result of requests from technology sponsors, the majority of which were medical devices companies, although 3 analyses were conducted following requests by clinicians and/or clinical departments from the hospital.

The modeling analyses were performed at different stages in the development of the technologies, from ‘idea screening,’ through ‘concept development,’ to the ‘pre-market phase’ to ‘market access.’ The authors note that some researchers may not consider the final phase to constitute ‘early modeling,’ but I accept their view that this stage still precedes any formal modeling presented to authorities in an official reimbursement submission. The main finding is that none of the assessments resulted in a firm ‘go/no-go’ decision about the technologies concerned, since none demonstrated that the technology could never be cost-effective. However, the assessments were helpful in gaining an insight into the technology’s potential cost-effectiveness in its intended context by informing further development or implementation. These insights could include the positioning of the technology (eg, position in the clinical pathway, or suitability for different patient sub-groups), or the need for additional research.

Therefore, there are two, interlinked, modeling efforts that could be performed. The first is the modeling of the potential cost-effectiveness of the product, viewed from the perspective of the external decision-maker(s) that will partly determine the market access for the technology. The second is a financial modeling effort, from the perspective of the company, to assess whether the potential financial returns will justify the investments in developing the product.

## Value of theGrutterset al Study

The main value of the study by Grutter et al is that, since the analyses were performed by an independent organization, the findings could be placed in the public domain, following some restrictions to preserve confidential findings on the technologies concerned. This is important, since although much has been written about the potential value of early health economic modeling, there are few published examples of its impact or value. This is because the vast majority of analyses have been conducted in-house by technology manufacturers (mainly pharmaceutical companies), where there is little need or incentive to make them public. The closest we see to actual examples relate to the preparatory work conducted by manufacturers to support ‘early engagement’ discussions with regulators and reimbursement authorities.^2^

## Issues for Further Discussion

Although the paper by Grutters et al makes a strong case for the role of early health economic modeling, there are other issues meriting discussion, should we wish to assess how useful early modeling could be. The first issue relates to the question of go/no-go decisions. It is correct to argue that if all the assessments conclude that a technology is cost-effective, it is hard to argue that it should be abandoned. But it is not clear how the assessments undertaken considered the price (or acquisition cost) of the technologies concerned. Some of the analyses conducted close to market access presumably included a price, but it is not clear whether the analyses conducted in earlier stages of development accounted for the manufacturer’s price expectations, or if any were even articulated. In the absence of inclusion of any price, or if price was varied in a sensitivity analysis, the modeling could still give the manufacturer an indication of whether particular price expectations could be met.

The point is that, whatever the benefits in improved health and cost savings, any technology could be rejected on grounds of lacking cost-effectiveness if the manufacturer’s price expectations were too high. Ideally, the manufacturer’s price expectations would be set early on and revised upwards or downwards as more information about the technology’s performance, or the need for additional research, becomes known. However, in most cases, decisions about price are usually discussed quite late in the development process, when arguably the decision might mainly be based on recovery of as many of the research and development costs as possible, rather than the level of profit that the technology is likely to make overall. Therefore, in order to best interpret the results of modeling, price expectations should be set earlier and reset periodically based on the acquisition of new information.

Secondly, as Grutters et al note, early health economic modeling can be useful in guiding future research into the technology concerned. This is often because of the need to obtain more accurate estimates of the key parameters of the model, but could also be because the model indicates that there may be benefits from studying the technology in new patient populations or at a different position in the treatment pathway.

Grutters et al are a little sceptical about whether probabilistic sensitivity analysis is the best way of characterizing uncertainty in situations where the quality of the information about the new technology is poor. Rather, they favour the use of deterministic sensitivity analysis. There is debate about this issue in the health economics literature, although one of the arguments in favour of a probabilistic approach is that it facilitates the use of formal value of information (VoI) analysis to guide future research. For example, VoI analysis can provide an estimate of the overall value of conducting more research to reduce decision uncertainty. It can also identify which model parameters it would most important to estimate more precisely. In addition, as Rothery et al^3^ point out, VoI analysis provides the manufacturer with a formal approach for considering the trade-off, at different stages of development, between carrying out further research and revising price expectations for the technology downwards. This links back to the point about pricing expectations made earlier.

Thirdly, one of the interesting features of the paper by Grutters et al is that it demonstrates that early health economic modeling can be performed at different time points in the development of a technology. In the paper, the time points were determined by the timing of the requests for analyses by the technology’s sponsor. In two cases the analysis was performed twice, although it is not clear whether this was at different time points or not. However, in principle, early stage health economic modeling is not a ‘one-time’ activity, but should be continuous and iterative, with the modeling being updated as more information becomes available, either about the technology itself or the environment in which it would be used (eg, emergence of new technologies, changes in prices, etc).^4^

For example, the price of the existing technology, that the manufacturer’s technology seeks to replace, could fall, making the new technology less attractive. This happened with drug-eluting stents in the United Kingdom. The price of bare metal stents fell, causing the incremental cost-effectiveness of drug-eluting stents to rise above the acceptable threshold in the United Kingdom.^5^ Alternatively, a new competitor technology could emerge, or there could be a change in decision-makers’ requirements for evidence on effectiveness or cost-effectiveness.

## Towards a Comprehensive Role for Early Stage Modeling

Grutters et al should be congratulated on an important contribution to the debate about the value of early health economic modeling. Based on their findings and the issues raised above, one could argue for a more comprehensive role for early stage health economic modeling. First, it would be iterative, with modeling being performed at multiple points in the development of the technology, normally at key points where either (*i*) an important decisions about the need for further research, or a change in positioning or pricing expectations needed to be made, or (*ii*) there was an important change in the external environment affecting the likely success or value of the technology.

Secondly, the modeling effort would comprise three, interlinked efforts (*i*) cost-effectiveness modeling from the perspective of the intended payer or reimbursement authority; (*ii*) modeling of the future research strategy for the technology, based on VoI analysis where possible; and (*iii*) financial modeling, of expected research costs, technology price and revenue, from the perspective of the manufacturer.

## Ethical issues

Not applicable.

## Competing interests

Author declares that he has no competing interests.

## Author’s contribution

MFD is the single author of the paper.
